# Trends in Microbiology 2024

**DOI:** 10.3390/life15010065

**Published:** 2025-01-08

**Authors:** Pabulo Henrique Rampelotto, Milan Kolář

**Affiliations:** 1 Bioinformatics and Biostatistics Core Facility, Institute of Basic Health Sciences, Federal University of Rio Grande do Sul, Porto Alegre 91501-970, Brazil; 2 Department of Microbiology, Faculty of Medicine and Dentistry, Palacky University and University Hospital Olomouc, 779 00 Olomouc, Czech Republic

## 1. Introduction

Microbiology is a key component of modern science, significantly influencing various fields, such as agriculture, medicine, and environmental management, particularly through the One Health approach, which recognizes the interconnectedness of human, animal, and environmental health. These innovations provide promising solutions to persistent challenges, including pest management, soil fertility enhancements, and the mitigation of antimicrobial resistance (AMR).

As the world confronts the challenges of climate change and food insecurity, integrating microbial agents into agricultural practices emerges as a critical strategy within the One Health framework. These agents enhance crop yields while promoting ecological balance, thereby reducing the need for chemical fertilizers and pesticides, which can have detrimental effects on both human health and the environment. Sustainable agricultural practices support food security and mitigate the environmental impacts associated with conventional farming methods.

Understanding the interactions between microbial agents and human health is crucial for developing innovative therapeutic strategies. Recognizing the interconnectedness of agriculture, human health, and environmental sustainability fosters a holistic perspective that enhances disease management and prevention. The synergy between agricultural microbiology and medical research underscores the importance of collaborative efforts to address global health challenges, ensuring that ecosystems and human populations thrive.

As we navigate the complex challenges posed by climate change, food insecurity, and emerging health threats, the need for a holistic understanding of microbiology is critical. The Special Issue “Trends in Microbiology 2024” was designed to reflect this urgency, aligning as a strategic and integrated project with the Section Microbiology of Life.

This Editorial highlights the 30 articles featured in this Special Issue, which are organized into two categories: biotechnology and health-related studies.

## 2. Biotechnology and Microbial Applications

The first part of the Special Issue explores the diverse potential of microbial applications across various fields.

Biocontrol efficacy: Heikal et al. [1] conducted an assessment of silver nanoparticles synthesized by *Trichoderma asperellum*, revealing their significant impact on the germination of *Hordeum vulgare*. This study underscores the potential of using biogenic nanoparticles as environmentally friendly alternatives to chemical pesticides.

Cholesterol management: Ziarno et al. [2] explored the cholesterol uptake by microflora from selected kefir starter cultures, suggesting that specific microbial strains can play a role in dietary interventions aimed at managing cholesterol levels, thus contributing to cardiovascular health.

Soil fertility enhancements: The work by Thu et al. [3] characterized novel potassium-dissolving purple nonsulfur bacteria isolated from alluvial upland soil. These bacteria can enhance soil fertility, promoting sustainable maize cultivation by improving nutrient availability.

Microbial dynamics: Bomer and Leverett [4] investigated the growth characteristics of a *Desmodesmus* species and its short-term impact on soil microbial dynamics. Their findings highlight how algal species can influence soil microbiomes, potentially leading to improved soil health and crop productivity.

Antibiotic resistance solutions: Issac Abraham et al. [5] identified *Ocimum sanctum* as a source of quorum-sensing inhibitors, which can combat AMR in both human and aquaculture pathogens. This discovery opens new avenues for the development of natural antimicrobial agents.

Fermentation processes: Khurshid et al. [6] reported enhanced citric acid production through the use of *Aspergillus niger*, utilizing sugarcane molasses in fermentation studies. This study demonstrates the economic viability of microbial fermentation processes in producing valuable industrial compounds.

Ecological implications: Christova [7] examined the association of *Phytophthora polonica* with alder decline in Bulgaria, emphasizing the ecological implications of microbial interactions in forest ecosystems.

Microbial diversity: Vasiliauskienė et al. [8] reported changes in the bacterial communities of biocomposites with different flame-retardants, emphasizing the importance of microbial diversity in material science applications.

Bioremediation insights: Bint-e-Zahira et al. [9] explored the presence of the *Trichoderma* species in industrial wastewater, presenting the morphological and molecular insights obtained from isolates. Their study contributes to the understanding of how specific fungi can be employed in bioremediation efforts to clean contaminated environments.

Biocontrol potential: Pereira et al. [10] assessed the biocontrol potential of *Serratia marcescens* and *Bacillus* species isolated from urban mangroves. Their findings highlight the effectiveness of these microbes in controlling plant pathogens, contributing to sustainable agricultural practices.

Microbial interactions: Rawi et al. [11] studied the antifouling potential of ethyl acetate extracted from *Pseudomonas aeruginosa*, indicating the role of marine bacteria in preventing biofouling in various environments.

Hyaluronic acid production: Abdullah Thaidi et al. [12] developed an *in situ* product recovery system using Amberlite IRA67 for the enhanced biosynthesis of hyaluronic acid by *Streptococcus zooepidemicus*, showcasing the potential of microbial fermentation in producing high-value biopolymers.

Toxicity studies: Ma et al. [13] investigated the toxicity of sugarcane wax metabolites to silkworms, highlighting the implications of plant–microbe interactions in agricultural pest management.

## 3. Health and Disease Studies

The second part of the Special Issue explores the impact of microbiology in health and disease.

Infectious disease complexity: Mihu et al. [14] reported a severe case of *Plasmodium falciparum* malaria in a Caucasian woman returning from Nigeria. This case illustrates the complexities of infectious diseases and the need for continued vigilance in monitoring emerging pathogens.

Gut microbiota and cancer: Rumyantsev et al. [15] discussed how gut microbiota influence gastrointestinal cancers through mechanisms involving obesity and chronic inflammation. This research suggests that microbial composition may serve as a biomarker for cancer risk and progression.

Antimicrobial resistance patterns: The study by de Souza et al. [16] evaluated the antimicrobial resistance patterns of *Pseudomonas aeruginosa* strains isolated from COVID-19 patients. Their findings reveal the ongoing challenges in managing infections, particularly in immunocompromised populations.

Emerging endemic zones: Khuroo et al. [17] presented a case series on alveolar echinococcosis in Kashmir, an emerging endemic zone. Their work highlights the importance of understanding regional epidemiology and the role of environmental factors in disease transmission.

Microbial dysbiosis and reproductive health: Wei et al. [18] explored the mechanisms behind male reproductive sterility triggered by the dysbiosis of intestinal microorganisms. This study emphasizes the intricate connections between microbiota and reproductive health, suggesting potential therapeutic targets.

Drug discovery approaches: Li et al. [19] conducted a structure-based virtual screening for potential inhibitors of *Mycoplasma pneumoniae*, showcasing the role of computational methods in identifying novel antimicrobial agents. Their findings contribute to the development of targeted therapies against resistant strains.

Real-world evidence: Sansone et al. [20] evaluated the effectiveness of imipenem/cilastatin/relebactam as a treatment for complicated bacterial infections, providing real-world evidence to inform clinical decision-making.

Dental caries and risk factors: Alzahrani and Bhat [21] focused on the prevalence of dental caries and associated risk factors among disabled individuals in institutional rehabilitation centers. These findings emphasize the importance of addressing health disparities and ensuring equitable access to oral health services, ultimately promoting better health outcomes for disabled individuals.

Oral microbiota and recovery: Todorić et al. [22] investigated the impact of pericoronary microbiota composition on recovery after third molar alveotomy, demonstrating the significance of oral microbiota in surgical outcomes.

Chagas disease mechanisms: Rossi et al. [23] explored the host–*Trypanosoma cruzi* interaction in Chagas disease, providing insights into the complex interaction between pathogens and host immune responses. 

Sarcocystis detection: Prakas et al. [24] conducted a study on the detection of three *Sarcocystis* species in blood samples from rodents, emphasizing the need to monitor zoonotic infections.

Antiparasitic efficacy: Albogami [25] evaluated the antiparasitic, antihepatotoxicity, and antioxidant efficacy of quercetin and chitosan against *Giardia lamblia* infection, highlighting potential therapeutic strategies for parasitic diseases.

Demodex co-infestations: Pyzia et al. [26] examined co-infestations of the *Demodex* species and culturable microorganisms in patients with blepharitis, shedding light on the complex interactions in the skin microbiome.

UV-C inactivation: Foschi et al. [27] assessed the ability of an ultraviolet C lamp to inactivate airborne microorganisms in a healthcare environment, emphasizing the importance of environmental sanitation.

Methicillin resistance: Khongsri et al. [28] compared the susceptibility of methicillin-resistant and methicillin-susceptible *Staphylococcus pseudintermedius* to empirical cotrimoxazole for canine pyoderma, addressing challenges in veterinary medicine.

Influenza characterization: Agustiningsih et al. [29] performed a molecular characterization of the influenza A/H3N2 virus isolated from Indonesian pilgrims, contributing to the understanding of viral evolution and epidemiology.

Circadian cycle: Amando et al. [30] explored the diurnal oscillation of the gut microbiota through the microbial culture, emphasizing the significance of temporal factors in microbiota studies.

## 4. Concluding Remarks

The current trends in microbiology illustrate the discipline’s expanding influence across various research domains. The integration of microbial applications in biotechnology allows for innovative strategies to address environmental and health-related challenges. Understanding the complex interactions between microbiota and human health remains imperative, as these relationships significantly impact disease outcomes. Continued research in these areas will enhance our understanding of microbial functions and facilitate the development of novel therapeutic and agricultural solutions.
